# First assessment of weeks-to-negative processing fluids in breeding herds after a Senecavirus A outbreak

**DOI:** 10.1186/s40813-023-00353-7

**Published:** 2024-01-05

**Authors:** Guilherme Preis, Neal R Benjamin, Deborah Murray, Emily Byers Taylor, Samuel Copeland, Grant Allison, Cesar A Corzo

**Affiliations:** 1 Veterinary Population Medicine Department, College of Veterinary Medicine, University of Minnesota, Saint Paul, MN USA; 2 The Program in Ecology, Evolution and Conservation Biology, University of Illinois Urbana- Champaign, Urbana-Champaign, IL USA; 3 New Fashion Pork, Jackson, MN USA; 4 Formerly employed at Prestage Farms, Clinton, NC USA; 5 Prestage Farms, Clinton, NC USA; 6 Walcott Veterinary Clinic, Walcott, IA USA

## Abstract

Senecavirus A (SVA) causes vesicular disease in swine and has been responsible for a rampant increase in the yearly number of foreign animal disease investigations conducted in the United States. Diagnostic investigations for SVA are typically performed by sampling animals individually, which is labor-intensive and stressful. Developing an alternative aggregate sampling method would facilitate the detection of this virus at the population level. In a preliminary study, SVA was detected in processing fluids (PF) collected in a breeding herd before and after outbreak detection. The objective of this study was to estimate the average number of weeks PF remain SVA-positive after an SVA outbreak. Ten farrow-to-wean breeding herds volunteered to participate in this studyby longitudinally collecting PF samples after an SVA outbreak was detected and submitting samples for RT-rtPCR testing. The PF samples from the 10 farms were SVA-positive for an average of 11.8 weeks after the outbreak. Here, we show that testing of PF may be a cost-effective method to detect SVA and help halt its spread in SVA-endemic regions.

## Background

Senecavirus A (SVA) has been responsible for swine vesicular disease outbreaks in different parts of the world [1–6], causing concern due to its clinical similarity to foot-and-mouth disease (FMD). Since all vesicular disease cases in pigs must be differentiated from FMD—a World Organization for Animal Health-listed disease—SVA can be costly for governmental animal health agencies and local swine industries. In the United States (US), SVA has been linked to a major increase in the yearly number of foreign animal disease investigations (FADI) since 2016 [7]. Aside from vesicular lesions, the virus has also been associated with increased neonatal mortality and diarrhea in piglets [1–3]. However, several aspects of the epidemiology, pathogenesis, immunology, and production impacts of this disease are poorly understood.

Diagnostic investigations for SVA are commonly performed by sampling animals individually by collecting oral, nasal, and rectal swabs [8], vesicular fluid, blood [2], and other tissue samples [2, 9]. However, individually sampling animals is labor-intensive and stressful; therefore, it is not optimal for disease monitoring and surveillance. The development of alternative aggregate sampling methods, such as oral fluids and processing fluids (PF) has facilitated the detection of pathogens at the population level [10, 11]. PF samples are comprised of the serosanguinous fluid recovered from piglet processing (i.e., castration and tail docking) during the first week after birth [12]. This sampling method is commonly used in commercial swine production to detect systemic viral pathogens, including porcine reproductive and respiratory syndrome virus (PRRSV) [11, 13]. Additionally, its use has been suggested to help establish the disease status of breeding herds undergoing the elimination of wild-type viruses [14]. Currently, there is scarce information regarding the presence of SVA in PF after an outbreak.

A preliminary study reported the presence of SVA RNA in PF 11 days before farm staff detected clinical signs suggestive of vesicular disease in one breeding farm [15]. In that study, the last SVA-positive PF sample was detected over 50 days after the outbreak was initially reported. This sustained detection of SVA RNA in processing fluids may be linked to long-term disease transmission. Characterizing the detection of SVA in PF would advance the epidemiological knowledge of this disease, potentially leading to the development of a systematic approach for breeding herd status classification, as is currently done with PRRSV [14].

The primary objective of this study was to estimate the average number of weeks PF remain SVA-positive after an SVA outbreak. As a secondary objective, we aimed to assess the production losses associated with an SVA outbreak.

## Methods

### Study design and breeding herd eligibility criteria

A cohort of 10 breeding sow farms undergoing an SVA outbreak was conveniently selected to participate in this study. Sow farms from commercial swine production companies or farms managed by veterinary clinics in the US were invited to participate in this study. Farm enrollment was dependent on their acceptance.

### Number of selected farms

The sample size of 10 sow farms was chosen to have 95% confidence for estimating the average number of weeks-to-negative processing fluids with a margin of error of 1.25 weeks, considering a standard deviation of 2 weeks-to-negative.

### Sample and data collection

All samples were collected by farm personnel according to protocols being utilized in pre-existing disease surveillance and monitoring programs. Farm staff were asked to collect up to four PF samples and store the samples in a -20 °C freezer until shipment, with a maximum of 50 sows/litters being represented per PF in any given week. Farm staff were asked to collect PF every two weeks for the first four months after the outbreak was detected. For months 5 and 6 after the outbreak, farm staff were asked to collect once per month—totaling 10 sampling weeks per farm in 6 months. PF samples collected before the outbreak was detected were also requested for testing when available. All samples were shipped to the University of Minnesota’s Veterinary Diagnostic Laboratory (UMN VDL) for testing.

All farms were asked to summarize if herds underwent a planned exposure process and share their production records electronically for data analysis.

### Laboratory testing and classification of SVA status

All PF samples were tested for the presence of SVA RNA by reverse-transcription real-time PCR (RT-rtPCR). RNA was extracted using a commercial extraction kit (Ambion MagMAX-96 viral RNA isolation kit; Life Technologies) and a magnetic particle processor (MagMAX Express-96 magnetic particle processor; Applied Biosystems), following the manufacturer’s guidelines. Although samples being assessed with RT-rtPCR are typically considered positive when the cycle threshold (Ct) values are 35.99 or lower and suspect when between 36 and 40 [16], we chose to classify any week as positive if at least one sample collected during that week had a Ct value under 40. Due to the decrease in sensitivity caused by aggregating multiple litters in one PF sample (due to a dilution effect) and since the prevalence of SVA-affected litters could be low (further decreasing sensitivity), suspect samples were treated as positives.

### Statistical analysis

Descriptive statistics from PF testing and the mean number of weeks-to-negativity with a 95% confidence interval were calculated. The 95% confidence interval for the average number of weeks-to-negativity was calculated with a one-sample t-test using the statistical software R [17] and the “mosaic” package [18]. Furthermore, production data shared by participating farms was graphically and descriptively summarized to assess the production losses.

## Results

### General farm information, characteristics, and outbreak occurrence date

A total of 10 farrow-to-wean breeding herds volunteered to participate in our study: seven belonged to one production system, one from a second production system, and the other two farms were managed by veterinary clinics (Table 1).

**Table 1:** Senecavirus A affected farms characteristics and information on the number of processing fluid (PF) samples and weeks tested

Farm ID	Farm Size (No. of sows)	Month and year of SVA outbreak	No. of collected samples	No. of weeks tested before the outbreak	No. of weeks tested after the outbreak	Follow-up time (No. of weeks)	No. of weeks tested	Average No. of samples per tested week
1	6,000	Jun-19	85	3	26	30	20	4.3
2	6,000	Sep-20	64	6	23	30	30	2.1
3	2,000	Aug-20	22	2	19	22	22	1.0
4	2,000	Aug-20	19	2	19	22	19	1.0
5	2,000	Aug-20	22	3	18	22	22	1.0
6	2,000	Aug-20	22	2	19	22	22	1.0
7	2,000	Aug-20	21	3	17	21	21	1.0
8	2,000	Sep-20	22	5	16	22	22	1.0
9	2,000	Oct-20	18	1	16	18	18	1.0
10	2,600	Nov-20	15	0	18	16	15	1.0
Total	—	—	310	—	—	—	—	—
Average	—	—	—	2.7	19.1	22.5	22.1	1.4

One sow farm detected the SVA outbreak in June/2019, five farms in August/2020, two in September/2020, one in October/2020, and the last in November/2020 (Table 1). The month of SVA outbreak detection was defined as the month when vesicular disease signs were first observed, and local animal health regulatory authorities conducted an FADI to rule out FMD.

### PF testing results

A total of 310 PF samples were tested across all 10 participating farms. The number of PF samples tested per farm ranged from 15 to 85. Farms 1 and 2 had an average of 4.3 and 2.1 PF samples tested per tested week, while all others had only one tested PF sample per week (Table 1). The overall follow-up time ranged from 16 to 30 weeks, averaging 22.5 weeks per farm (Table 2).

**Table 2:** Summary statistics of the number of tested weeks and SVA status in processing fluids over time for 10 sow farms undergoing an outbreak

Statistic	Minimum value	First Quartile	Median	Third Quartile	Maximum value	Average	Standard Deviation
Total follow-up time in weeks	16	21.2	22	22	30	22.5	4.5
No. of followed weeks before outbreak detection	0	2	2.5	3	6	2.7	1.8
No. of followed weeks after outbreak detection	16	17.2	18.5	19	26	19.1	3.1
Last positive week after outbreak detection	1	9.5	11.5	14.7	21	11.8	5.2
Number of negative weeks between positive weeks	1	1.2	2	3	10	2.9	3
Number of negative weeks after the last positive week	2	4.2	6	8.5	18	7.3	5.2

**Fig. 1:**
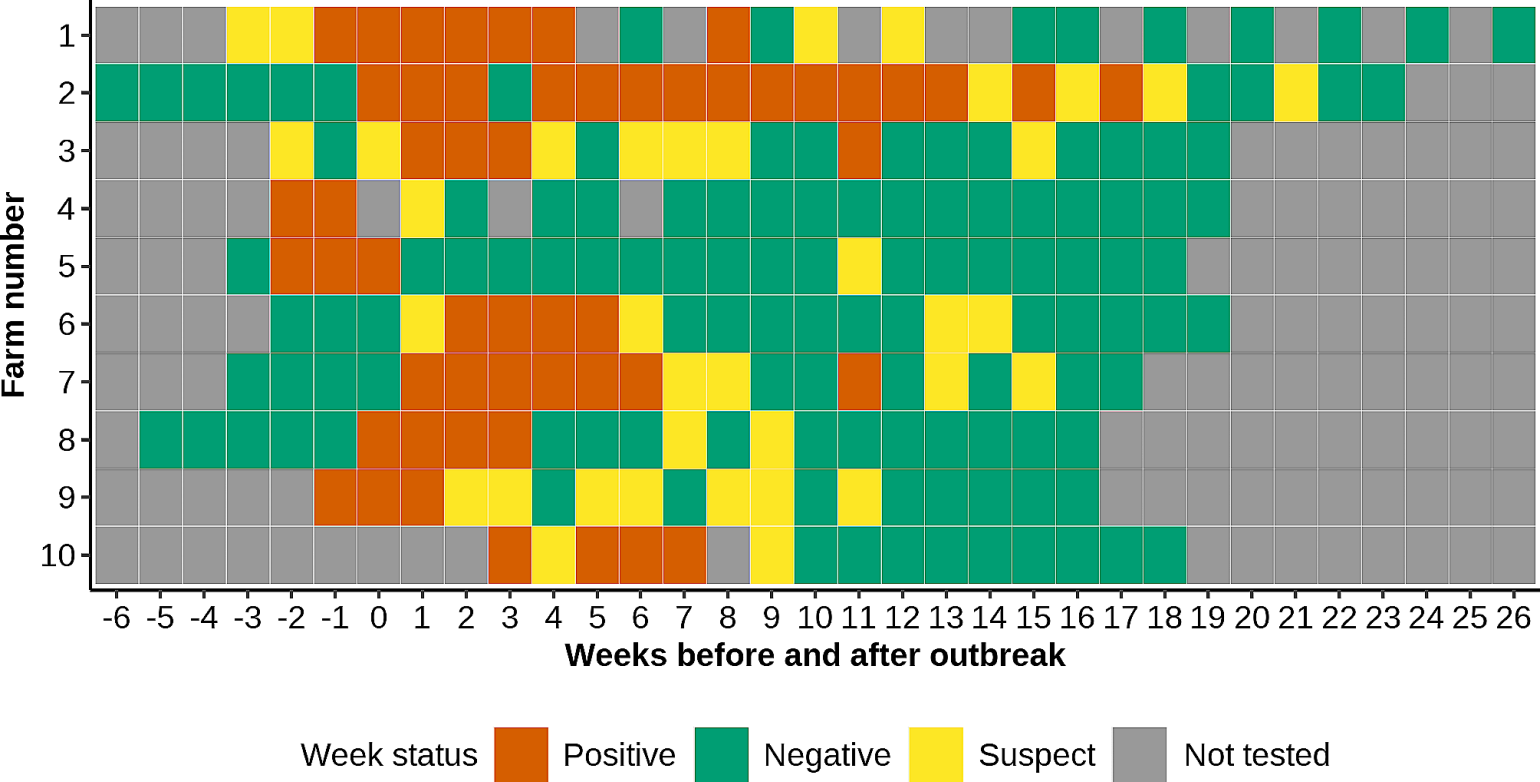
Weekly PF SVA status in 10 sow farms before and after outbreak detection. Red = At least one PF sample had an RT-rtPCR Ct value below 36. Yellow = At least one PF sample had an RT-rtPCR Ct from 36 to 40. Green = All tested samples were negative. Grey = No samples were tested. The suspect results were considered positive in this study, but they are shown here to visualize the variation in results over time

**Fig. 2:**
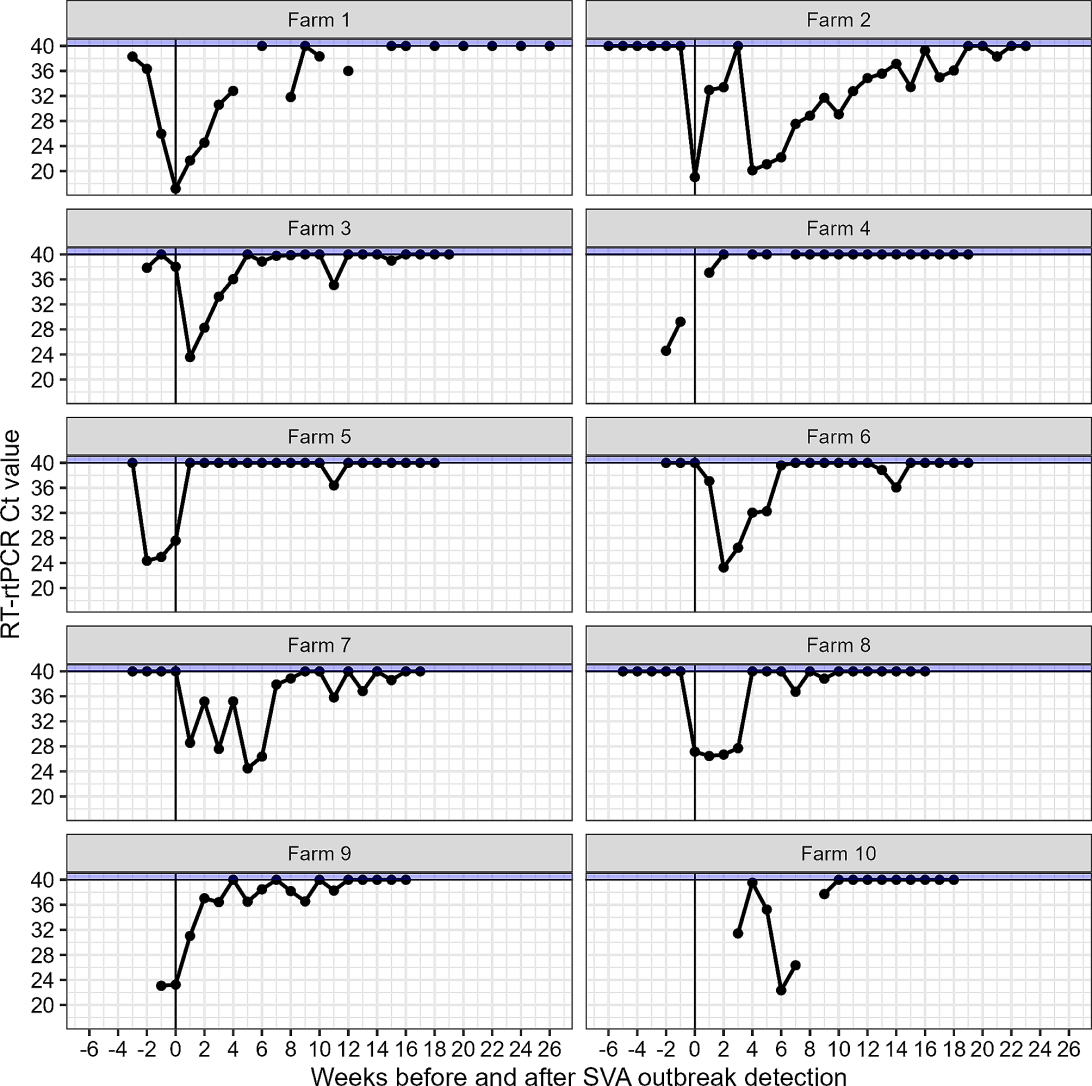
Weekly processing fluids SVA RT-rtPCR results by farm, before and after SVA outbreak detection. The reported Ct values are the results from the positive sample with the lowest Ct value for any given week. Samples with a Ct = 40 (blue shaded area) represent a week where all tested processing fluid samples were negative

Five sow farms had SVA-positive PF present before clinical signs were evident to the farm staff, with the earliest detection up to three weeks before the FAD investigation (Figs. 1 and 2). The number of SVA-positive weeks post-outbreak ranged from 1 to 21, with an average of 11.8 (95% CI– 8.1, 15.5) weeks across all ten sow farms.

A wide range (1 to 10 weeks) of consecutively negative weeks between positives was observed (Table 2; Fig. 1). There was also a wide range of weeks with negative results after the last positive, ranging from 2 to 18.

### Farm-level interventions to control SVA

Aside from the required FAD investigation performed in all 10 farms, only one farm (Farm 2) reported interventions in response to the outbreak. In this farm, mass exposure was attempted eight weeks after the outbreak detection, followed by herd closure to elicit herd immunity and decrease within-herd transmission. Briefly, the farm veterinarian collected the vesicular fluid from SVA-affected animals and mixed it with 500mL of phosphate-buffered saline (PBS) solution. The solution was kept at -20 °C, and an aliquot was sent to the UMN VDL for SVA RT-rtPCR testing, which was positive with a Ct value of 17. The SVA solution was then thawed, and sows, gilts, and heat-check boars were intranasally exposed via nasal cannula using 0.1mL of the solution. All animals that did not develop vesicular lesions or did not share the same pen with an affected animal were inoculated. The remaining SVA solution was used to spray the pen area where the gilts were kept.

### Impact of the SVA outbreaks on production parameters

The only marked difference in production parameters was the weekly pre-weaning mortality (PWM) (proportion of pigs that died in each cohort of weaned piglets) during the four weeks following the outbreak in Farm 1. The mean PWM for the 52 weeks preceding the outbreak was 13.9%; however, PMW increased from 9.1 to 18.1 and 23% during the initial three weeks after clinical signs were identified. The PWM peaked during the fourth week at 42.7% and returned to baseline values observed before the outbreak around five weeks after the initial detection of the outbreak.

Farms 3, 4, 5, 6, 7, 8, and 9 also shared their production data, but no marked differences were seen after the onset of the outbreaks. Production data from Farms 2 and 10 was not available.

## Discussion

The use of aggregate samples for pathogen herd-level assessment is often promoted as a tool to aid during the control, monitoring, and disease elimination efforts. This study provides evidence that testing PF samples for the presence of SVA is a valuable and efficient tool for monitoring, surveillance, and determining herd-level SVA status. PF samples are commonly collected for PRRSV detection in sow farms [11, 13]; therefore, swine veterinarians can readily use PF to possibly detect SVA before the farm staff detects clinical signs. To the authors’ knowledge, this is the first study to report the use of PF samples to detect SVA and estimate the number of weeks where PF samples remain positive during an outbreak.

We detected SVA-positive PF samples up to three weeks before clinical signs were observed on five farms. It has previously been reported that SVA can be detected in individual-level samples—such as oral and nasal secretions, feces, and serum—1 to 3 days prior to clinical onset [8, 19–21], indicating that pigs may be infectious before the incubation period is over. Interestingly, our results suggest that clinical signs may take weeks to be detected after SVA is present and being shed in a sow farm, potentially resulting in further undetected within and between-herd transmission and disease prevalence if contaminated materials and infected pigs are being unknowingly moved to different sites. We hypothesize that this longer delay in detecting vesicular disease signs at the herd level could be due to a low disease prevalence in the weeks preceding the outbreak detection, making it harder to see vesicle-affected animals. In addition, clinical signs may not be apparent in all cases, further limiting detection by farm workers when walking barns to assess health. The decreasing trend of the Ct values from weeks − 3 to 0 in Farm 1, as shown in Fig. 2, might be due to a combination of increasing viremia and shedding levels of recently infected sows and the prevalence of SVA-affected litters, which would increase the concentration of SVA RNA in the pooled PF samples. Conversely, the continuous increase in Ct values in the following weeks could be due to decreased viremia levels and the prevalence of SVA-affected litters, diluting the total SVA RNA within the pooled PF samples. However, we note that no fixed number of litters is represented in each PF sample tested in this study.

It is currently unknown how the dilution effect from litter aggregation and pooling affects SVA RT-rtPCR results and sensitivity. One previous study reported the detection of PRRSV in PF when only one PRRSV-positive pig was present in an aggregate sample of 50 litters or approximately 600 pigs [22]. Another study estimated the probabilities of PF samples testing positive to PRRSV to be 43%, 80%, and 95% when a single PRRSV-positive piglet was present among 784, 492, and 323 PRRSV-negative piglets, respectively [13]. Even though such findings support the high sensitivity of the currently available molecular diagnostic tools, no similar study has been published with SVA. Further work is needed to assess the effect of dilution on sensitivity since this might affect the interpretation of results from farms in later stages of infection or with a previous recent history of SVA exposure, where the proportions of positive litters are expected to be lower.

The average number of weeks until the last SVA-positive PF was detected was 11.8 (95% CI– 8.1, 15.5) and ranged from 1 to 21 weeks (Farm 4 and Farm 2, respectively). It is unknown whether previous SVA exposures may have affected the reported results. Previous SVA exposure may elicit some level of herd immunity that could potentially shorten virus transmission and the weeks-to-negative PF. We could not ascertain previous SVA exposure in any of the tested farms.

Interestingly, only one farm (Farm 2) attempted mass exposure and herd closure to eliminate the disease eight weeks after the outbreak was detected. There is a strong possibility that mass exposure contributes to the sustained transmission of SVA, which may explain the prolonged detection of SVA in PF on this farm. The larger number of sows in inventory also may have contributed to this prolonged SVA detection, as the time needed for SVA to transmit to most animals within a population could be longer on larger farms. However, Farm 1 had an equal number of sows in inventory, tested a larger number of weekly samples than all other farms, and yet did not test as consistently positive on weekly testing as Farm 2. Therefore, more extended shedding periods may be expected when SVA elimination is attempted through mass exposure methods.

It is still unclear how many consecutive weeks of SVA RT-rtPCR-negative testing are required to achieve optimal confidence that SVA is not being transmitted within the farrowing house. Sow farms in this study had a variable number of consecutive negative weeks between positive results, ranging from 1 to 10 weeks (Table 2). The sporadic detection of a pathogen during weekly monitoring using PF samples has also been reported for PRRSV, where the pathogen was detected after 11 consecutive weeks of negative PF results [23]. These findings should be considered if a consistent weaning of SVA-negative piglets is desired.

The number of weeks that farms tested negative after the last positive test result ranged from 2 weeks on two farms (Farms 2 and 7) to 18 weeks on Farm 4 (Fig. 1), with an average value of 7.3 (Table 2). However, Farm 4 showed atypical results as the last SVA-positive PF was detected at week 1 after the outbreak. We are uncertain why this farm achieved apparent PF-negative status so quickly. However, one possibility is that prior herd immunity could have played a role, as has been previously reported for PRRSV [24]. Additionally, the differences in follow-up time between farms likely contributed to the different number of negative weeks after the last positive since positive results could have appeared with further testing.

Given that Farm 5 tested PF-positive again after 10 consecutive negatives, we note that 10 consecutive negative weeks may not be enough to consider a farm PF-negative. Regardless, a comprehensive individual animal sampling scheme should be implemented at this time to increase the probability of SVA detection at lower prevalences. Further work is needed to identify potential alternative sampling schemes after PF-negativity.

Farms 1, 3, 4, 5, 6, 7, 8, and 9 shared production data from before and after the SVA outbreak, but only Farm 1 displayed marked differences. Previous health issues in Farms 3–9 may have masked the effects of the SVA outbreaks. It is also possible that genetic differences present in diverse strains of SVA are responsible for different clinical outcomes (e.g., higher or lower incidence of neonatal diarrhea) across outbreaks. PWM from Farm 1 reached 42.7% in the third week after outbreak detection, quickly returning to baseline values from before the outbreak. This result is consistent with what has been previously reported [3]. Neonatal mortality has been commonly reported in some farms during an SVA outbreak, with young piglets displaying clinical signs of lethargy, weakness, diarrhea, and sudden death, with pre-weaning mortality as high as 70% [1, 3, 25–28]. The mechanisms that cause the reported mortalities are not fully understood.

The results of this study are limited to the 10 participating breeding farms, which may not be enough to accurately describe the variability of weeks-to-negative PF. Notably, not all farms could sample and test with the same frequency throughout the entire study period, which may have impacted our findings.

Furthermore, we do not have any evidence to recommend how many weekly samples—and the number of litters aggregated in each sample—are needed to determine SVA presence in a farm accurately. Therefore, further studies are needed to address this question.

## Conclusions

This is the first study reporting the use of PF samples to detect and monitor SVA. Practicing veterinarians may expect sow farms to detect SVA-positive PF samples for an average of 11.8 weeks after an outbreak. In the face of an outbreak, we suggest that sow farms should monitor the herd through PF until at least ten consecutive RT-rtPCR-negative weeks are achieved if weaning SVA-negative piglets is desired, given that one of the farms in this study tested positive after 10 negative weeks. Nonetheless, further studies need to be performed to identify other sampling strategies to implement after consistent PF-negativity to have high confidence in weaning SVA-negative piglets. Sow farms at high risk of transmitting SVA and high-health herds such as genetic nuclei and multipliers should consider testing PF for SVA presence and potentially detect the virus even before farm staff notices the clinical signs. Routine PF testing can enhance biosecurity and decrease the risk of spread to other farms.

## Data Availability

The raw data supporting the conclusions of this article will be made available by the authors upon reasonable request.
